# Correction: Trust in science during the COVID-19 pandemic: A typology of internet users in South Africa

**DOI:** 10.1371/journal.pone.0344001

**Published:** 2026-02-26

**Authors:** Anne Reif, Lars Guenther, Monika Taddicken, Peter Weingart

The affiliation for the first author is incomplete. Anne Reif is not only affiliated with #1 but also with #4: Technische Universität Braunschweig, Braunschweig, Germany.
