# Supplementary material for: Trust in science during the COVID-19 pandemic: A typology of internet users in South Africa
Source: PLoS One. 2026 Jan 28;21(1):e0340881. doi: 10.1371/journal.pone.0340881 (PMC12851501; doi:10.1371/journal.pone.0340881)
Supplement: S1 Appendix — (DOCX) [file pone.0340881.s001.docx]

**S1 Appendix**

Supplemental material to manuscript

“Trust in science during the COVID-19 pandemic: A typology of internet users in South Africa”

**Table A.** Model comparison information for confirmatory factor analysis.

| Models |  |  |  | |  | |  | |  | |  |  | |  | | Model comparison with five factors | | |  |
| --- | --- | --- | --- | --- | --- | --- | --- | --- | --- | --- | --- | --- | --- | --- | --- | --- | --- | --- | --- |
| Total sample | *χ²* | | *df* | *χ²/df* | | AIC | | SSA-BIC | | RMSEA[90% CI] | CFI | | TLI | | SRMR | *χ²* diff. | *df* diff. | *p* |  |
| One factor | 882.07 | | 54 | 16.33 | | 44650.05 | | 44700.11 | | .10[.10, .11] | .91 | | .89 | | .05 | 542.38 | 10 | <.001 |  |
| Two factors (E+I, B+T+D) | 664.85 | | 53 | 12.54 | | 44434.83 | | 44486.98 | | .09[.08, .10] | .94 | | .92 | | .04 | 325.16 | 9 | <.001 |  |
| Four factors (E, I, B, T+D=O) | 475.70 | | 48 | 9.91 | | 44211.55 | | 44318.27 | | .08[.07, .09] | .96 | | .94 | | .03 | 136.01 | 4 | <.001 |  |
| Five factors (E, I, B, T, D) | 339.69 | | 44 | 7.72 | | 44127.67 | | 44198.59 | | .07[.06, .08] | .97 | | .95 | | .03 |  |  |  |  |
| Note*. n*= 1,426. AIC ­= Akaike Information Criterion; BIC =­ Bayesian Information Criterion; SSA =­ Sample Size Adjusted; RMSEA ­= Root Mean Square Error of Approximation; CFI = Comparative Fit Index; TLI = Tucker-Lewis Index; SRMR =­ Standardized Root Mean Square Residual; E = Expertise; I = Integrity; B = Benevolence; T = Transparency; D = Dialogue; O = Openness (Besley et al., 2021); two-factor solution based on exploratory factor analysis. | | | | | | | | | | | | | | | | | | | |

**Table B.** Standardised estimates per dimension of trust in science.

| Items | Scientists can be trusted because they… | Standard. estimates (std. al) | *R²* estimates |
| --- | --- | --- | --- |
| Exp1 | … are real experts in their particular fields.^1^ | .761*** | .579 |
| Exp2 | … rarely make mistakes.^1^ | .598*** | .358 |
| Exp3 | … check each other’s results before publishing them.^2^ | .746*** | .557 |
| Int1 | … adhere to strict rules and standards in their work.^1^ | .811*** | .658 |
| Int3 | … do not adjust their results for strategic and financial reasons, or to please others’ expectations.^1^ | .774*** | .599 |
| Ben1 | … work for the common good.^1^ | .855*** | .731 |
| Ben3 | … would not purposely bring harm to others.^3^ | .766*** | .587 |
| Tra1 | … regularly inform the public about relevant and important results of their research. | .805*** | .648 |
| Tra2 | … do not conceal details of their research. | .751*** | .564 |
| Tra3 | … explain scientific information in a comprehensible way. | .813*** | .661 |
| Dia1 | … listen to public opinions on their topics and research.^1,4^ | .797*** | .635 |
| Dia2 | … do not shy away from public discourse and participation. | .834*** | .696 |
| *Note*. *n*=1,426, ****p*<.001. 5-point rating scale from 1 ‘strongly disagree’ to 5 ‘strongly agree’. All items based on Reif and Guenther (2022), ^1^based on Wissenschaft im Dialog (2021), ^2^based on Ipsos MORI (2014).  ^3^based on Mayer and Davis (1999), ^4^based on Schäfer and Metag (2019). | | | |

**Table C.** Zero-order Correlation matrix for all group identifying variables (dimensions of trust) with trust in science on different levels and willingness to be vulnerable.

|  | 1 | 2 | 3 | 4 | 5 | 6 | 7 | 8 | 9 | 10 |
| --- | --- | --- | --- | --- | --- | --- | --- | --- | --- | --- |
| **Dimensions of trust^1^** |  |  |  |  |  |  |  |  |  |  |
| 1. *Expertise* | 1 |  |  |  |  |  |  |  |  |  |
| 1. *Integrity* | .62*** | 1 |  |  |  |  |  |  |  |  |
| 1. *Benevolence* | .61*** | .63*** | 1 |  |  |  |  |  |  |  |
| 1. *Transparency* | .62*** | .66*** | .70*** | 1 |  |  |  |  |  |  |
| 1. *Dialogue* | .54*** | .55*** | .63*** | .69** | 1 |  |  |  |  |  |
| **Levels of trust^2^** |  |  |  |  |  |  |  |  |  |  |
| 1. Trust in science | .52*** | .47*** | .48*** | .51*** | .45*** | 1 |  |  |  |  |
| 1. Trust in universities | .53*** | .49*** | .48*** | .52*** | .44*** | .61*** | 1 |  |  |  |
| 1. Trust in priv. companies/industry | .52*** | .49*** | .51*** | .53*** | .45*** | .55*** | .71*** | 1 |  |  |
| 1. Trust in scientists | .55*** | .62*** | .54*** | .55*** | .49*** | .66*** | .74*** | .67*** | 1 |  |
| 1. Willingness to be vulnerable^1^ | .52*** | .45*** | .58*** | .59*** | .59*** | .48*** | .38*** | .44*** | .46*** | 1 |

*** *p* ≤ .001 (one-tailed), *n*=1,555 - 1,610. Variables in italics were used to identify the groups in a latent profile analysis. ^1^ 1 ‘strongly disagree’ to 5 ‘strongly agree’, ^2^ 1 ‘do not trust at all’ to 5 ‘trust a great deal’.

**Table D.** Fit indices of latent profile analysis (1-5 profiles).

| Profiles | AIC | AWE | BIC | CLC | KIC | Entropy | Prob. min | Prob. max |
| --- | --- | --- | --- | --- | --- | --- | --- | --- |
| 1 | 21881 | 22036 | 21934 | 21863 | 21894 | 1 | 1 | 1 |
| 2 | 18894 | 19143 | 18979 | 18863 | 18913 | .85 | .95 | .96 |
| 3 | 17894 | 18237 | 18011 | 17851 | 17919 | .86 | .89 | .95 |
| 4 | 17555 | 17992 | 17705 | 17501 | 17586 | .81 | .86 | .92 |
| 5 | 17567 | 18098 | 17748 | 17500 | 17604 | .75 | 0 | .91 |

ACI = Akaike information criteria, AWE = Approximate weight of evidence, BIC = Bayesian information criterion, CLC = Classification Likelihood Criterion, KIC = Kullback information criterion.


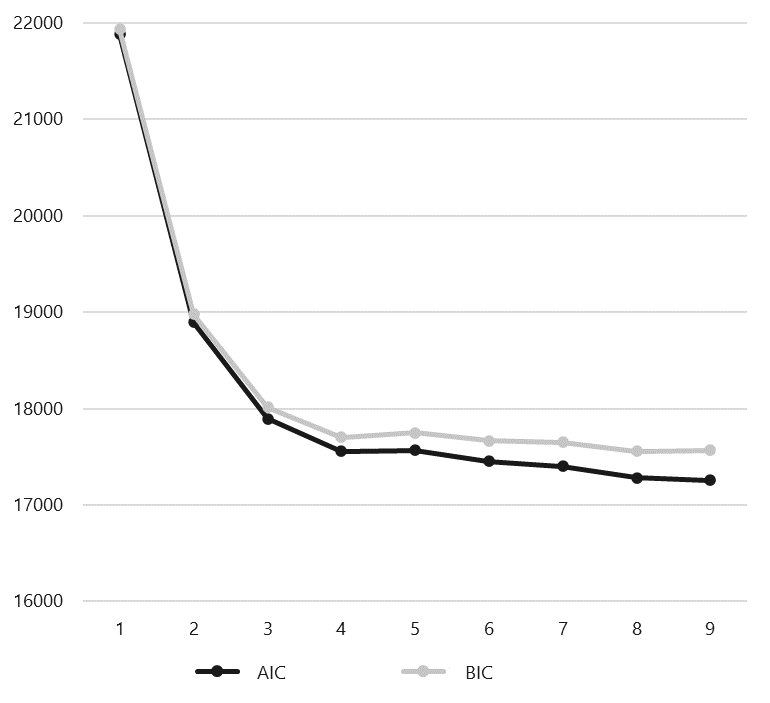


**Figure A.** Elbow graph for Akaike information criteria (AIC) and Bayesian Information Criterion (BIC).

**Table E.** Description of four SA groups of trust (*M* (*SD*)).

|  | 1 Fully trusting  (*n*= 465; 30%) | 2 Highly trusting  (*n*= 532; 35%) | 3 Moderately trusting  (*n*= 428; 28%) | 4 Rather untrusting  (*n*= 116; 8%) | *F*(3, 1526-1537) | η² |
| --- | --- | --- | --- | --- | --- | --- |
| **Levels of trust^1^** |  |  |  |  |  |  |
| Macro: science | **4.62 (.60)’** | 4.11 (.80)’ | 3.52 (.89)’ | 2.91 (1.08)’ | 211.61*** | .29 |
| Meso: university | **4.70 (.60)’** | 4.17 (.81)’ | 3.63 (.89)’ | 2.90 (1.16)’ | 143.51*** | .30 |
| Meso: private companies/industry | **4.57 (.60)’** | 3.97 (.89)’ | 3.37 (.94)’ | 2.43 (1.03)’ | 193.10*** | .33 |
| Micro: scientists | **4.64 (.65)’** | 4.08 (.80)’ | 3.40 (.90)’ | 2.58 (1.06)’ | 192.22*** | .37 |
| **Dimensions of trust^2^** |  |  |  |  |  |  |
| *Expertise* | ***4.55 (.47)’*** | *3.77 (.63)’* | *3.21 (.63)’* | *2.36 (.83)’* | *217.80**** | *.54* |
| *Integrity* | ***4.66 (.47)’*** | *3.84 (.70)’* | *3.06 (.76)’* | *1.91 (.70)’* | *329.23**** | *.60* |
| *Benevolence* | ***4.72 (.43)’*** | *3.95 (.63)’* | *2.92 (.68)’* | *1.86 (.69)’* | *388.72**** | *.68* |
| *Transparency* | ***4.63 (.40)’*** | *3.70 (.53)’* | *2.85 (.53)’* | *1.66 (.51)’* | *391.20**** | *.76* |
| *Dialogue* | ***4.57 (.61)’*** | *3.63 (.76)’* | *2.76 (.76)’* | *1.74 (.63)’* | *379.21**** | *.59* |
| **Willingness to be vulnerable^2^** | **4.20 (.91)’** | 3.42 (.95)’ | 2.70 (.88)’ | 1.78 (.78)’ | 265.83*** | .39 |
| **Frequency of contact with science^3^** |  |  |  |  | *F*(3, 1424-1532) | η² |
| Direct contact |  |  |  |  |  |  |
| Science centres, museums or planetariums | **3.83 (1.18)^a,b,c^** | 3.29 (1.15)^a,d,e^ | 2.93 (1.18)^b,d^ | 2.61 (1.23)^c,e^ | 58.07*** | .10 |
| Zoos, aquariums, nature reserves or botanical gardens | **3.73 (1.14)^a,b,c^** | 3.20 (1.06)^a^ | 3.03 (1.10)^b^ | 2.96 (1.13)^c^ | 36.57*** | .07 |
| Science events, such as science festivals, science cafés, public lectures and expert discussions | **3.82 (1.23)’** | 3.27 (1.17)’ | 2.92 (1.26)’ | 2.41 (1.22)’ | 61.81*** | .11 |
| Conversations with scientists | **3.28 (1.46)’** | 2.76 (1.27)’ | 2.35 (1.23)’ | 1.90 (1.01)’ | 55.01*** | .10 |
|  |  |  |  |  | *χ^2^(df, n)* | *V* |
| Met scientist in person^4^ | **65%** | 61% | 50% | 55% | *χ^2^(6, 1541)* = 26.81*** | .09 |
| Working in science |  |  |  |  | *χ^2^(6, 1541)* = 34.16*** | .11 |
| Yes, currently | **16%** | 11% | 9% | 12% |  |  |
| No, but I used to | **22%** | 20% | 15% | 7% |  |  |
| No, never | 62% | 69% | 77% | **81%** |  |  |
|  |  |  |  |  | *F*(3, 1424-1532) | η² |
| Contact via social agents (conversations with others, such as family, colleagues or friends) | **3.96 (1.08)^a,b,c^** | 3.56 (1.03)^a,d,e^ | 3.21 (1.02)^b,d^ | 2.96 (1.07)^c,e^ | 51.23*** | .09 |
| Contact via (journalistic) media |  |  |  |  |  |  |
| TV | **4.29 (.93)’** | 3.97 (.97)’ | 3.59 (1.07)’ | 3.24 (1.16)’ | 54.66*** | .10 |
| Radio | **3.67 (1.13)’** | 3.12 (1.11)’ | 2.83 (1.04)’ | 2.49 (1.15)’ | 59.72*** | .11 |
| Printed newspapers | **3.63 (1.13)’** | 3.24 (1.10)’ | 2.93 (1.02)’ | 2.53 (1.20)’ | 45.60*** | .08 |
| Printed magazines | **3.64 (1.16)’** | 3.23 (1.16) ^‘^ | 2.92 (1.04)’ | 2.58 (1.20)’ | 43.46*** | .08 |
| Journalistic online sources | **4.03 (1.03)^a,b,c^** | 3.55 (1.03)^a,d,e^ | 3.19 (1.04)^b,d^ | 3.02 (1.09)^c,e^ | 59.44*** | .11 |
| Non-fiction books | **3.52 (1.20)^a,b,c^** | 3.20 (1.19)^a,d^ | 3.09 (1.19)^b,e^ | 2.51 (1.22)^c,d,e^ | 24.22*** | .05 |
| Fictional content | **4.18 (.99)^a,b,c^** | 3.93 (1.04)^a,d,e^ | 3.74 (1.12)^b,d^ | 3.52 (1.26)^c,e^ | 18.74*** | .04 |
| Homepages of scientific institutions | **4.30 (.89)^a,b,c^** | 3.87 (1.04)^a,d,e^ | 3.64 (1.09)^b,d^ | 3.49 (1.26)^c,e^ | 37.39*** | .07 |
| Contact via social media |  |  |  |  |  |  |
| Blogs and forums | **4.02 (1.00)’** | 3.62 (1.01)’ | 3.34 (1.05)’ | 2.95 (1.06)’ | 49.51*** | .09 |
| Wikis | **4.21 (1.01)^a,b,c^** | 3.83 (1.01)^a,d^ | 3.59 (1.07)^b^ | 3.55 (1.13)^c,d^ | 30.36*** | .06 |
| Video platforms | **4.34 (.91)^a,b,c^** | 3.92 (1.00)^a,d^ | 3.78 (1.05)^b^ | 3.63 (1.13)^c,d^ | 30.19*** | .06 |
| Social networking sites | **3.99 (1.09)^a,b,c^** | 3.50 (1.07)^a,d,e^ | 3.24 (1.04)^b,d^ | 3.09 (1.15)^c,e^ | 43.55*** | .08 |
| Twitter | **3.66 (1.26)^a,b,c^** | 3.08 (1.19)^a,d,e^ | 2.80 (1.15)^b,d^ | 2.49 (1.15)^c,e^ | 48.59*** | .09 |
| Messenger apps | **3.53 (1.27)^a,b,c^** | 2.94 (1.25)^a,d,e^ | 2.69 (1.16)^b,d^ | 2.41 (1.09)^c,e^ | 47.06*** | .09 |
| Podcasts | **3.54 (1.26)’** | 2.94 (1.19)’ | 2.67 (1.12)’ | 2.25 (1.09)’ | 54.41*** | .10 |
| Search engine use | **4.42 (.88)^a,b,c^** | 4.07 (.98)^a,d,e^ | 3.85 (1.09)^b,d^ | 3.60 (1.21)^c,e^ | 33.89*** | .06 |
| Online participation (index) | **3.65 (1.03)’** | 2.98 (.94)’ | 2.64 (.88)’ | 2.21 (.86)’ | 118.40*** | .19 |
| **Trust in^2^:** |  |  |  |  | *F*(3, 1428-1521) | η² |
| Direct contact | **4.69 (.66)’** | 4.33 (.84)’ | 3.69 (1.11)’ | 3.00 (1.35)’ | 143.52*** | .23 |
| Contact via social agents | **3.71 (1.10)’** | 3.18 (1.02)’ | 2.94 (.94)’ | 2.63 (1.13)’ | 58.25*** | .10 |
| Contact via journalistic media | **3.87 (1.11)’** | 3.15 (1.07)’ | 2.82 (1.02)’ | 2.37 (1.09)’ | 100.63*** | .17 |
| Contact via social media | **3.63 (1.22)’** | 3.02 (1.12)’ | 2.63 (1.00)’ | 2.09 (1.07)’ | 90.53*** | .15 |
| **Other variables** |  |  |  |  | *F*(3, 1424-1533) | η² |
| Trust in politics^1^ | **3.03 (1.52)’** | 2.45 (1.28)’ | 2.00 (1.06)’ | 1.41 (.76)’ | 75.65*** | .13 |
| Trust in the media^1^ | **3.66 (1.18)’** | 3.06 (1.09)’ | 2.60 (1.02)’ | 2.03 (1.03)’ | 105.93*** | .17 |
| Trust in religion/the church^1^ | **4.08 (1.21)^a,b,c^** | 3.68 (1.22)^a,d^ | 3.48 (1.26)^b,d^ | 3.45 (1.50)^c^ | 19.95*** | .04 |
| Trust in the military^1^ | **3.77 (1.19)’** | 3.11 (1.16)’ | 2.75 (1.11)’ | 2.15 (1.17)’ | 89.21*** | .15 |
| Trust in other people in general^1^ | **3.50 (1.25)^a,b,c^** | 2.92 (1.05)^a,d,e^ | 2.52 (.92)^b,d^ | 2.24 (.98)^c,e^ | 79.45*** | .14 |
| Interest in science (Index)^5^ | **4.49 (.75)’** | 4.15 (.75)’ | 3.81 (.89)’ | 3.47 (1.11)’ | 75.40*** | .13 |
| Knowledge (Index)^6^ | **4.31 (.79)^a,b,c^** | 3.73 (.78)^a,d,e^ | 3.25 (.80)^b,d^ | 3.03 (.86)^c,e^ | 162.15*** | .24 |
| Reservations (Index)^2^ | **3.61 (1.05)^a,b,c^** | 3.38 (.91)^a,d^ | 3.19 (.89)^b,d^ | 3.25 (.82)^c^ | 15.62*** | .03 |
| Promises (Index)^2^ | **4.48 (.68)’** | 4.03 (.75)’ | 3.57 (.85)’ | 3.01 (1.06)’ | 151.38*** | .24 |
| Whenever science and religion conflict, religion is always right.^2^ | **3.45 (1.55)^a,b^** | 3.17 (1.42)^a^ | 2.96 (1.44)^b^ | 3.08 (1.57) | 8.04*** | .02 |
| **Socio-demographics** |  |  |  |  | *F*(3, 1537) | η² |
| Age | **35.26 (10.90)^a^** | 33.34 (11.12)^a^ | 33.75 (11.72) | 34.10 (11.28) | 2.58 | .01 |
| Religiosity | **4.05 (1.18)^a,b,c^** | 3.79 (1.18)^a^ | 3.60 (1.27)^b^ | 3.64 (1.43)^c^ | 10.56*** | .02 |
|  |  |  |  |  | *χ^2^(df, n)* | *V* |
| Gender (female) | 57% | 53% | **58%** | 52% | *χ^2^(3, 1541) =* 3.28 | .05 |
| Population group |  |  |  |  | *χ^2^(9, 1541) =* 32.06*** | .08 |
| Black | **82%** | 73% | 68% | 70% |  |  |
| Coloured | 8% | 10% | **11%** | 8% |  |  |
| Indian/Asian | 2% | 3% | **5%** | 2% |  |  |
| White | 8% | 13% | 16% | **21%** |  |  |
| Geographical location |  |  |  |  | *χ^2^(15, 1541) =* 17.18 | .06 |
| Metropolis | **24%** | 21% | 20% | 22% |  |  |
| City | 34% | **38%** | 36% | 29% |  |  |
| Town | 24% | 26% | **28%** | **26%** |  |  |
| Rural area | 18% | 15% | 16% | **22%** |  |  |
| Level of education |  |  |  |  | *χ^2^(15, 1541) =* 10.48 | .07 |
| I never attended school | 0% | 0% | 0% | 0% |  |  |
| Primary school | 1% | 1% | 0% | **2%** |  |  |
| Grade 9/GET phase | **4%** | 3% | 3% | 4% |  |  |
| Matriculation certificate | 27% | 34% | **36%** | 29% |  |  |
| College | 24% | 19% | 19% | **27%** |  |  |
| Tertiary education (University) | **44%** | 43% | 42% | 38% |  |  |
| Political orientation |  |  |  |  | *χ^2^(6, 11326) =* 52.57*** | .14 |
| Liberal | 26% | 22% | **28%** | 27% |  |  |
| Moderate | 25% | 40% | **45%** | 38% |  |  |
| Conservative | **49%** | 38% | 27% | 36% |  |  |

*Note. n*= 1,541. *M* = mean, *SD* = standard deviation. Variables in italics were used for latent profile analysis. ^1^ 1 ‘do not trust at all’ to 5 ‘trust a great deal’, ^2^ 1 ‘strongly disagree’ to 5 ‘strongly agree’, ^3^ 1 ‘never’ to 5 ‘very often’, ^4^ ‘yes’ or ‘no’, ^5^ 1 ‘not interested at all’ to 5 ‘very interested’, ^6^ 1 ‘know nothing’ to 5 ‘know a great deal’, **Chi-squared* tests and *F*-values significant at *p*≤.05, ***p*≤.01, ****p*≤.001. Groups sharing the same superscripts differ in the Bonferroni post-hoc test on *p*≤.05 per variable. If all groups significantly differ, it is indicated by **’**. Numbers in bold indicate the highest numbers per variable.

References

Besley, J. C., Lee, N. M., & Pressgrove, G. (2021). Reassessing the Variables Used to Measure Public Perceptions of Scientists. *Science Communication*, *43*(1), 3–32. https://doi.org/10.1177/1075547020949547

Ipsos MORI. (2014). *Public Attitudes to Science 2014: Main Report*. https://www.ipsos.com/sites/default/files/migrations/en-uk/files/Assets/Docs/Polls/pas-2014-main-report-accessible.pdf

Mayer, R. C., & Davis, J. H. (1999). The Effect of the Performance Appraisal System on Trust for Management: A Field Quasi-Experiment. *Journal of Applied Psychology*, *84*(1), 123–136.

Reif, A., & Guenther, L. (2022). How representative surveys measure public (dis)trust in science: A systematisation and analysis of survey items and open-ended questions. *Journal of Trust Research.* Advance online publication. https://doi.org/10.1080/21515581.2022.2075373

Schäfer, M. S., & Metag, J. (2019). *WissensCHaftsbarometer Schweiz 2019.* Universität Zürich. https://wissenschaftsbarometer.ch/wp-content/uploads/2019/10/Tabellenband_Wissenschaftsbarometer_2019_DE-1.pdf

Wissenschaft im Dialog. (2021). *Wissenschaftsbarometer 2021*. https://www.wissenschaft-im-dialog.de/fileadmin/user_upload/Projekte/Wissenschaftsbarometer/Dokumente_21/WiD-Wissenschaftsbarometer2021_Broschuere_web.pdf
