# Supplementary material for: Trust in science during the COVID-19 pandemic: A typology of internet users in South Africa
Source: PLoS One. 2026 Jan 28;21(1):e0340881. doi: 10.1371/journal.pone.0340881 (PMC12851501; doi:10.1371/journal.pone.0340881)
Supplement: Attachment — Submitted filename: Response to Reviewers.docx [file pone.0340881.s003.docx]

**Response to Reviewers**

Dear reviewers, dear editors,

We would like to express our sincere gratitude to the reviewers for their detailed, insightful, and constructive comments, which have significantly contributed to the enhancement of our manuscript entitled: “Trust in science during the COVID-19 pandemic: A typology of internet users in South Africa.” We also wish to congratulate the editors on assembling highly qualified experts to review our work.

We have taken considerable time to carefully and thoroughly revise the entire manuscript, addressing all reviewer suggestions and concerns with great attention. Due to personal circumstances, we required additional time to ensure that all issues were properly and adequately addressed. We are confident that these extensive revisions have substantially improved the quality and clarity of our study.

In particular, we have revised the entire introduction and discussion sections, added a new and detailed last chapter to transparently discuss important limitations, and updated the relevant literature to reflect recent developments. We elaborated on the cultural specifics of the South African public and expanded our discussion on the theoretical implications of our findings for, thereby clarifying and strengthening the contribution of our research. Additionally, we included more detailed information on our methods, conducted supplementary analyses, revised parts of the results section, and addressed all formatting, style, open science, and ethics requirements. The data are now openly available on OSF to promote transparency and reproducibility.

We would like to thank the editors and reviewers once again for their valuable and supportive feedback. We hope that with these revisions, our manuscript now aligns with the journal’s standards and is suitable for publication.

In the following, we will respond to each reviewer comment in detail.

**Detailed response to Reviewer #1**

Reviewer #1: Dear authors,
thank you very much for providing such an interesting manuscript, that I really appreciated to review. The manuscript focuses on the identification of subgroups within the South African population with diverging levels of trust in science measured by different reasons to trust science. Based on recent literature, the authors derive a five factors solution for measuring trust in science. They identify four distinct subgroups among their sample, that mainly differed in their levels on trust in science but had comparable patterns of reasons to trust and on other variables (e.g. contact with science through different mediators).

In its present form, I would not recommend the manuscript for acceptance. I propose that major revisions be undertaken.

We thank Reviewer #1 for their thoughtful and constructive feedback on our manuscript. We appreciate the recognition of the relevance and interest of our research. The comments provided valuable guidance for the necessary major revisions to enhance the manuscript.

The current version of the manuscript attempts to incorporate too many different thematic aspects, resulting in the loss of a clear focus early on. It remains unclear to the reader what the main objective of this paper is: differentiating between online and offline users, comparing the ZA sample with findings from WEIRD states, developing a new instrument for measuring trust in science at the micro level, distinguishing between trust in science at the micro-, meso-, and macro-levels, classifying those who trust, empirically testing the theoretical model by Reif & Guenther (2022) (see Supplemental Figure 1), including mediation analyses of mediated contact with trust in science, or tracking changes in trust over the course of COVID-19?

The manuscript requires greater coherence and clarity, with a more precise focus on the most important research questions. While these points are individually interesting, attempting to empirically address all of them at once makes the analysis less comprehensible.

We sincerely appreciate the reviewer’s insightful comment, which has significantly helped us refine our manuscript. In response, we have revisited and revised the entire introduction to clarify the main focus of the paper. Our primary aims are now explicitly articulated as follows: (1) to identify and characterise trust groups within the specific cultural context of South Africa, particularly in relation to their trust in science; and (2) to explore variables associated with trust in science, especially regarding contact with science and (3) perceived changes in trust attributable to the COVID-19 pandemic. We also acknowledge that developing a new instrument for measuring trust was not a central aim of this study; therefore, references to this have been removed to maintain a clearer focus. We agree that the broad scope of themes previously addressed may have led to some lack of clarity, and we have taken steps to improve focus throughout the manuscript. The context of digitisation of science communication and the COVID-19 pandemic were intended to frame the study, rather than to serve as comprehensive thematic aims themselves. Once again, thank you for guiding us towards a more focused and coherent presentation.

**Content**
The authors describe their sample as “online users” and “digitized public”. Yet, it is not clear why the authors refer to this notion and how “online users” differ from “non-online users”. The authors should provide more information for the necessity of this distinction and cite literature that suggests fundamental differences between these subpopulations. While it is understandable that, due to the nature of the survey being conducted online, the participants should be online users, this alone does not seem sufficient to justify narrowing the perspective to such an extent.

Thank you for your valuable feedback. We would like to clarify that our intention was not to compare online users with offline users as distinct groups. Instead, we use the concept of the “digitised public” to frame the context of our study, reflecting the increasing importance of digital media in science communication. As the survey was conducted online, participants are characterised as part of this digital or “digitised” audience. We understand that this framing does not imply a strict dichotomy between online and offline groups; rather, we view the “digitised public” – or publics, as we examine sub-groups – to be hybrid. Individuals may engage with science through both online and offline contact. In the revised manuscript, we have further clarified this framing at the beginning and have toned down references to “online users” and “digitised publics” throughout the text. In the methods section, we explicitly state that the survey was conducted online. We discuss the limitations to it in the limitation section and provide reasoning for this choice, emphasising that this approach was necessary given the research context. We appreciate your input, which has helped us improve clarity.

Given that the scales are primarily designed to measure trust at the micro level, this should be appropriately reflected in the manuscript, with an emphasis on trust in scientists. Particularly in the theoretical background, the current wording gives the impression that trust is being measured at the macro level, where different reasons for trusting science are assessed (e.g. RQ1).

We recognize the reviewer’s impression. In response, we have revised the theoretical section to and have included additional information highlighting the importance of the micro-level for public trust in science. Furthermore, we have acknowledged the reviewer’s concern in the limitations section to clarify the scope of our measures and their focus on micro-level trust. We appreciate the input.

**Methodology**
a) For measuring trust in science (micro level), the authors employ newly developed scales, which are partly adapted from existing instruments and partly created anew. They indicate that the dimensions include expertise, integrity, benevolence, transparency, and dialogue. Given that these measures are pivotal for the classification of participants in the LPA, the authors should elaborate further on the measurement instrument. Especially, the issue of construct validity has not been sufficiently addressed and is not evident to the reader.

b) The authors also assess the factorial structure of the instrument through confirmatory factor analysis. Given that the discussion highlighted unresolved issues with item allocation, the authors might want to consider employing exploratory factor analysis for further evaluation. For example, the peer review item is classified under expertise, although it could be more fittingly associated with integrity, dialogue, or transparency as aspects of good scientific standards.

Finally, the authors should justify their decision to forgo the use of well-established scales (e.g., Hendriks et al., 2016; Ziegler, Ricarda, & Kremer, Bastian (2023). Wissenschaftsbarometer 2022 - Repräsentative Bevölkerungsumfrage zu Wissenschaft und Forschung in Deutschland. GESIS, Köln. ZA7641 Datenfile Version 1.0.0, https://doi.org/10.4232/1.14077 ) for assessing trust in scientists.

Once again, thank you for your important feedback. Since the primary aim of our study was not to develop and validate a new scale, we have largely based our measurement on the “Public Trust in Science Scale,” which has been recently tested and published, and which closely aligns with the dimensions we used, including expertise, integrity, benevolence, transparency, and dialogue orientation. We have added references to this scale and its foundational instruments to clarify its established validity. However, to address the concerns, we have discussed the measures and acknowledge in the discussion and limitations sections that different theoretical perspectives exist regarding the dimensions of trust. We believe these clarifications help situate our scales within the broader measurement landscape and address validity considerations.

c) The behavioural measures need to be critically revised. The item 'I would be willing to let scientists have complete control over the future of our society' appears particularly problematic, as it implies that scientists should be responsible for political decisions, unnecessarily politicising the issue. A better approach to capturing the behavioural aspect might be to focus on laypeople trusting and considering scientists’ recommendations in their individual decision-making processes.

Thank you very much for this insightful comment. We fully agree that the item in question may be problematic, as it could be interpreted as politicising the role of scientists. We appreciate this important point and, in response, we have discussed it as a limitation in the discussion section after discussing the item in light of the cultural context. We agree that future research should consider refining this item in particular to include scientists’ recommendations in personal decision-making contexts.

d) The authors utilise Latent Profile Analysis (LPA) as the classification method, which was conducted based on the levels of trust in science. There are a few points that the authors should briefly address in this regard: LPA estimates the probabilities for each individual to belong to a specific class, highlighting the probabilistic nature of this approach. However, in the manuscript, class memberships are treated as deterministic, thus overlooking the uncertainty in classification. The authors should explain the methodology they employed to arrive at the final solution, where each individual is assigned to exactly one class.

Thank you for this valuable observation. In the revised manuscript, we have included additional details about the Latent Profile Analysis (LPA). Specifically, we clarify that, for simplicity and interpretability, individuals were assigned to the class for which they have the highest posterior probability, effectively treating class membership as deterministic. We also acknowledge in the limitations section that alternative methodological approaches could have been employed and that different solutions regarding the number and nature of profiles are possible. Nonetheless, given the empirical data, the four-profile solution was the most appropriate. We appreciate this important point, which has helped us improve transparency regarding our classification process.

**Results**
The results imply that the groups differ only in the degree of trust they place in scientists for various reasons. This is also evident in the frequency and manner of their interaction with scientific information and their trust therein. When examining the findings altogether, they reveal intriguing yet sometimes contradictory patterns: for instance, class 1 demonstrates high levels of trust not only in scientists but also in politicians and the military. How can these findings be interpreted?

Thank you for highlighting this important aspect. In response, we have elaborated further on the nuanced differences observed among the groups, also acknowledging that differences in the patterns of dimensions were rather subtle. As a specific example, we have added the following statement to the discussion of results (p. 27, lines 544–547): “The groups of *rather untrusting* individual were found to distinguish the most between the levels; the *fully trusting* and *highly trusting* the least which may be a hint to their general high level of trust in authorities that is also reflected in their high trust in the military and politics.”

Furthermore, the overall results prompt two additional questions:
a) Are these findings truly reflective of real differences, or could they be influenced by response biases among participants? It would be advantageous if the authors addressed this more comprehensively and included further indicators of high data quality (e.g., excluding participants with low item variance across multiple variables).

Thank you for raising this important point. We acknowledge that response biases cannot be entirely ruled out as a limitation of our study. However, we believe that overall data quality is high, as evidenced by the respondents’ demonstrated attentiveness, particularly through their detailed responses to the open-ended questions. We have included this clarification in the manuscript to transparently acknowledge this limitation while also highlighting the indicators of data quality observed in our sample.

b) Since the results appear to indicate only subtle differences, how practical is the precise measurement of trust in science, scientists, and contact with scientific information for future research from a perspective of parsimony? Do these findings not strongly suggest that a single item could typically provide a sufficient, though possibly superficial, insight into the level of trust?

Thank you for raising this important question. As noted earlier, we have elaborated in the revised limitations section that the observed differences across the trust dimensions were indeed subtle and primarily reflected an overall gradient from highly trusting to less trusting groups. We also acknowledge that, given the subtle variations, the practical utility of measuring five distinct dimensions of trust compared to a single, superficial item can be questioned. Nonetheless, our findings suggest that a more nuanced measurement may still capture meaningful distinctions, even if subtle, which could be relevant depending on the research context.

The authors describe in their results section, that they performed ANOVAs to compare group means across different variables (between-groups). However, when providing more details about the different classes, the authors also refer to mean comparisons within the classes but across these variables (within-groups). It is not feasible for the reader to assess, whether these differences are of descriptive or inference statistical nature (e.g. Section 6.2).

We appreciate the thoughtful comment. We agree that the previous version of the manuscript did not provide sufficient detail regarding the analyses. In response, we have revised the methods section within the analysis chapter to include information on the post-hoc tests performed to compare group means. Additionally, we found merit in exploring within-group differences for the trust dimensions relevant to group differentiation, which is why we have conducted additional analyses. These are now included in the results section and summarised in Table 3. We thank the reviewer for this great idea.

**Discussion**
The authors are advised to significantly revise the discussion. It is difficult to discern a consistent line of reasoning, as the discussion often veers off, giving the impression that the authors are trying to include too much content in too little space. Moreover, the discussion includes aspects that are more appropriately discussed in other parts of the manuscript: a large portion of the commentary on the groups should be placed in the results section, and the correlations should ideally be introduced in the results section before being elaborated on in the discussion.

Thank you for this valuable and constructive feedback. While we wish to maintain a clear distinction between the presentation of results and their interpretation, we agree that the discussion can be made more focused and cohesive. In response, we have substantially revised the entire discussion section, dedicating more space to a more in-depth and organized reflection on the findings. The reviewers’ comments have been instrumental in guiding these improvements. We also acknowledge that the correlations should be introduced in the results section for clarity, and we have now incorporated this information accordingly. We believe these revisions enhance the clarity of the manuscript and ultimately its contribution to the state of research and science communication practice.

**Open Science**
In light of open science practices, the authors are requested to briefly state whether there was a citable preregistration for the project detailing the hypotheses and analysis steps. Additionally, for the publication, it would be useful to provide a link to the repository that will be made accessible after the project’s completion, which will allow readers to access the data, materials, and scripts.

Thank you for this important request. We have clarified that the survey and our exploratory approach were not preregistered in the methods section. However, we are pleased to provide the link to the repository, which will be made publicly accessible upon the publication of the paper.

**Language**
The authors are requested to carefully review the manuscript once more. Additionally, they should consider whether some of the statements are made with the appropriate level of rigor and whether the existing evidence supports them, e.g.:
- L29ff. “These surveys, however, mainly applied direct single-item measures of public trust in science that neglect the assumption that depending on cultural and individual beliefs and experiences, trust in science may be based on different reasons.”

Thank you for your honest and constructive feedback. We have carefully reviewed the entire manuscript to ensure that all statements are appropriately supported and articulated with the required rigor. Wherever necessary, we have strengthened our statements by referencing current relevant literature. As stated above, we have revised the entire introduction. Since the measurement of trust is not the central focus of our paper, we have also revised the specific sentence in question to clarify the important point. The sentence now reads: “However, these surveys do not account for sub-group differences, nor do they explore the various reasons underlying trust in science.” (p. 2, lines 41–42) In the revised manuscript, this statement is more clearly integrated with the current research literature, providing appropriate context and support.

- L53/L54 sentence structure

Thank you for pointing out the issue with the sentence structure. We have removed the original sentence in the introduction. To preserve the conceptual connection, we have included a reference to the idea of different notions of trust in science—such as blind trust and informed/cognitive trust—on the second chapter, which discusses the theoretical conceptualisation of trust (now p. 5–6, lines 118–124). This update helps frame the research gap and the formulation of our first research question more clearly.

- L118-120 sentence structure

Thank you for your valuable feedback. We have revised the sentence in lines 118–120 concerning the types of contact commonly considered by public surveys. The improved sentence has been relocated to lines 151–153 (p. 7) in the revised manuscript. We believe this revision significantly enhances the clarity and readability of the text. We appreciate your helpful suggestions.

- L544ff. “Unlike regression analyses, which typically examine predictors of trust in science, our methodological approach acknowledges the assumption that more trust in science is not necessarily better. Instead, it adopts a more value-neutral assumption that trust varies individually and the reasons for this variation are manifold.”

We have now included examples from the current literature of studies that employed regression analyses, to better contextualise our methodological approach and distinguish it from such analyses.

Others
- L161 – 4 / see also L233/234 (Source links)

Thank you for your careful review. We have corrected the issues related to the reference links as noted.

- L220 – RMSEA above .60?

Thank you for pointing out this error. It was an unintended mistake; the correct RMSEA value is above .06, not .60. We have now corrected this in the manuscript.

- Table 3., Table 4., Supplemental Table 5. – Last Column – “5”? Rather untrusting - should be “4”

Thank you for catching this error. We have corrected “5” to “4” in the last column of Tables 3, 4, and Supplemental Table 5 (now Table E in S1 Appendix).

In conclusion, I would like to thank the authors once again for this intriguing manuscript and hope that the points raised in this review are clear, comprehensible, and useful. I look forward to reading the final version of the manuscript.

Thank you very much for your kind and supportive words. We sincerely appreciate your thoughtful feedback and recommendations throughout the review process. Your comments have been instrumental in helping us improve the clarity, rigor, and overall quality of our revised manuscript.

**Detailed response to Reviewer #2**

The paper, “Trust in Science During the COVID-19 Pandemic: A Typology of Internet Users in South Africa,” explores trust in science among South African internet users during the COVID-19 pandemic. Using latent profile analysis, it identifies four groups with varying trust levels—fully trusting, highly trusting, moderately trusting, and rather untrusting. The paper examines trust dimensions (e.g., expertise, benevolence, transparency) and their relationships with contact types, such as direct engagement with scientists or social media. Trust patterns are shown to be influenced by socio-demographics, cultural factors, and personal experiences during the pandemic. The authors highlight the need for group-specific communication strategies to foster trust, particularly among groups with lower trust levels.
While the paper is relevant and engaging, the following revisions are necessary for it to be considered for potential publication in PLOS ONE:

Thank you for your detailed and constructive review of our manuscript. We appreciate your positive assessment of the relevance and engagement of our work, as well as your insightful suggestions for improvement which have been very helpful.

1. The introduction needs further development to provide clearer context and better integration of existing literature.

We acknowledge the reviewer’s observation regarding the need for further development of the introduction. Their impression matches reviewer #1’s comment. In response, we have thoroughly revised this section to provide clearer context and better integration of the relevant literature. Specifically, we have explicitly stated the primary objectives of our study: (1) to identify and characterise trust groups within the cultural setting of South Africa, with a particular focus on trust in science; (2) to examine variables related to trust, including contact with scientific information; and (3) to investigate perceived changes in trust during the COVID-19 pandemic. Furthermore, we have emphasised the role of digital science communication and the pandemic as framing elements of our research. We appreciate your guidance, which has helped us create a more focused and coherent introduction.

2. The results section requires clarification and more detailed explanations, particularly regarding latent profile analysis and trust dimensions.

We appreciate the reviewer’s suggestion and have revised the analyses section within the methods chapter to include additional details on the Latent Profile Analysis procedure. Additionally, in the Limitations section, we acknowledge that alternative methodological approaches could have been considered and that different solutions concerning the number and nature of the profiles are possible. In the results section, we have carefully re-evaluated the characterisation of the identified groups and provided more detailed explanations of how these groups differ from one another, enhancing clarity and interpretability in the discussion section.

3. The discussion section would benefit from enhanced theoretical integration, contextual specificity, and articulation of broader implications.

We thank the reviewer for their honest impression. In response, we have revised both the discussion section and the entire introduction to strengthen the theoretical integration and contextual specificity. We made particular efforts to align the discussion with the theoretical frameworks outlined earlier and updated the literature review to reflect the most current research developments. We believe that these revisions enhance the coherence of the discussion and effectively contextualise our findings within broader theoretical and practical implications, thus providing a more comprehensive conclusion to the paper.

Below are specific comments for further refinement:
1. Abstract: The concept of “infodemic” may not be easily understood by a lay audience. Consider providing a brief definition (e.g., an overabundance of information and misinformation related to COVID-19).

We concur with the reviewer’s suggestion. To enhance clarity for a lay audience, we have removed the initial sentence of the original abstract that mentioned the term “infodemic.” Additionally, we have revisited and refined the remaining content of the abstract to better emphasise the key findings and implications of our study.

2. Page 2: The discussion of group-specific approaches to understanding trust in science emphasizes the role of cultural and individual beliefs in shaping trust patterns. However, it overlooks relevant work by Borinca et al. (2024), which examines trust in science among individuals with varying trust levels (low vs. high) and incorporates both WEIRD (Irish) and non-WEIRD (Kosovo Albanian) samples. This paper experimentally manipulates social norms as a communication strategy and demonstrates how compliance with health advice influences trust, solidarity, and behavior during the COVID-19 pandemic.
o Including this reference would enhance the discussion by providing evidence on how tailored communication strategies and social norms impact trust in culturally diverse groups.
o Beyond the fact that your paper is cross-sectional while Borinca et al.’s is experimental, clarifying how your work uniquely contributes to this area—perhaps through a focus on specific cultural contexts or unique trust mechanisms—would strengthen the discussion.
Reference:
Borinca, I., Griffin, S. M., McMahon, G., Maher, P., & Muldoon, O. T. (2024). Nudging (dis)trust in science: Exploring the interplay of social norms and scientific trust during public health crises. Journal of Applied Social Psychology, 54(8), 487–504.

We are very grateful for this excellent suggestion. Accordingly, we have incorporated the suggested reference to Borinca et al. (2024), which now serves as a key citation in our paper – in particular in the introduction and discussion. In addition, to further strengthen our contextualisation, we have included additional relevant literature examining public trust in science across diverse cultural settings. These additions help to present a more comprehensive overview of the current research landscape and clarify how our study contributes uniquely—particularly through its focus on a group-specific approach in a specific cultural context. Overall, these revisions enhance the depth and breadth of the discussion, situating our work within the broader field more effectively.

3. South African Context: The discussion of trust and contact during COVID-19 lacks sufficient context specific to South Africa. Adding examples or background on trust dynamics in South Africa would provide a more comprehensive understanding, particularly as this is a non-WEIRD setting.

We sincerely appreciate this valuable feedback. We agree that the previous version of the manuscript did not adequately emphasise the unique cultural context of South Africa. In response, we have elaborated extensively on South Africa’s specific cultural dynamics throughout the entire revised manuscript. The new introduction now centrally addresses the cultural context, and each theoretical chapter includes additional references and descriptions relevant to South Africa. In the fully revised discussion section, we have dedicated a comprehensive paragraph to interpreting our results within the framework of South Africa’s distinctive cultural environment. We believe these revisions significantly enhance the contextual richness and overall contribution of our study. Once again, thank you for your insightful suggestion.

4. Theoretical Framework: The distinction between macro, meso, and micro levels of trust is a valuable framework. However, the categorization of “trust in scientists in general” as micro-level trust seems unclear. Typically, micro-level trust involves interpersonal trust in specific individuals, whereas trust in “scientists in general” appears more collective and might align better with meso or macro levels. Clarifying this would enhance the theoretical framework’s precision and interpretability.

Thank you for the valuable feedback. In response, we have revised the theoretical section to better clarify the categorisation of trust levels emphasising the significance of the micro-level in shaping public trust in science. Furthermore, we have incorporated the reviewer’s comment into the new limitations section, where we discuss how the measures primarily focus on micro-level aspects of trust.

5. Trust Profiles:
o The labels for the four groups (e.g., fully trusting, highly trusting) are somewhat simplistic. Could an individual be highly trusting in expertise but moderately trusting in transparency? Acknowledging potential overlaps or ambiguities would add depth to the findings.
o Specify how these profiles were quantitatively defined. Were the cutoffs for the trust levels determined based on a statistical criterion or subjectively labeled?

We appreciate the reviewer’s insightful feedback, which highlights an important limitation of our trust profiles. We have addressed this directly in the revised manuscript by adding a statement to the new limitations section (p. 32, lines 670–675): “Although variations in the patterns across dimensions were found, they were unexpectedly subtle as the tendency of all answers to the dimensions reflected the general descending order of trust groups from *highly trusting* to *rather untrusting*. Unlike expected, we did not identify groups of individuals highly trusting in expertise but moderately trusting in transparency, for instance. This is why somewhat simplistic labels were chosen for the four groups.”

Regarding the definition of these profiles, the comparison was primarily based on group means. We have further clarified this aspect and provided more detailed information about how the trust levels and profiles were quantitatively determined in the analyses section to enhance transparency and understanding.

6. Trust Dimensions: While the authors note differences in trust dimensions across groups, more elaboration is needed. For example, which dimensions (e.g., expertise, transparency, benevolence) most strongly influenced group distinctions?

We appreciate this excellent suggestion. In response, we conducted additional analyses of effect sizes for the mean differences across trust dimensions, which we have now included in the results section (chapter 6.1). These analyses reveal that transparency was the dimension with the most pronounced differences between groups, indicating that perceptions of transparency most strongly influenced the distinctions among trust profiles. We believe this addition provides valuable insights and enhances the interpretive depth and potential impact of our findings. In addition, we have elaborated a bit more on the general group differences.

7. Contact Types:
o The results indicate fewer individuals in the “moderately trusting” and “rather untrusting” groups had personal contact with scientists. Was personal contact measured as binary (e.g., yes/no) or continuous (e.g., frequency of interactions)?
o Were specific types of contact (e.g., formal talks, informal discussions) associated with higher levels of trust? Clarifying this would provide actionable insights for science communication strategies.

This is an important point. In response, we have added detailed descriptions of how the items “met scientist in person” and “working in science” were measured as requested. Additionally, we moved the descriptions and results related to these items from chapter 6.1 (additional variables) to chapter 6.2, and from Table 4 to Table E in the Supplementary Material (S1 Appendix) for clearer presentation. Our findings indicate that all forms of contact with science tend to be higher among groups with greater trust in science. We have also emphasised and discussed this relationship more prominently in the revised discussion section, highlighting its implications for science communication strategies.

8. Behavioral Trust Measures: The paper includes behavioral trust measures, such as willingness to rely on scientists’ efforts or allow them control over societal decisions. Were significant differences observed in these measures across the four groups? Highlighting these differences would reveal the behavioral implications of trust levels. If no differences were found, discussing why these measures did not vary would also be valuable.

We thank the reviewer for their honest opinion. The discussion section of the previous manuscript lacked structure and focus. We hope that through the revision of the entire discussion the important statement regarding behavioural trust measures will not be overlooked by readers. Specifically, on page 27 (line 547 and following), we discuss and interpret our findings, noting that higher trust in science across the different levels was associated with greater trust dimensions and an increased willingness to be vulnerable, which reflects behavioural trust.

9. Open-Ended Responses: The responses to RQ3 on changes in trust during COVID-19 are intriguing but lack clarity on how they were analyzed. Were these responses analyzed systematically (e.g., thematic analysis), or are they illustrative examples? Clarifying this would strengthen the methodological rigor.

We appreciate the reviewer’s important question. In response, we have revisited and expanded the description of the thematic analysis procedures within the methods section. We confirm that the examples presented are used illustratively to enhance the characterisation of the groups, rather than as the sole basis of analysis.

10. General Discussion:
o The general discussion effectively summarizes the findings but would benefit from explicitly linking the trust profiles to the macro, meso, and micro levels of trust in the theoretical framework.
o Additionally, the discussion should address how the paper’s findings contribute to or challenge existing theories of trust in science.

We sincerely appreciate your valuable suggestions. In response, we have revised the General Discussion to explicitly link the identified trust profiles more clearly to the macro, meso, and micro levels of trust within our theoretical framework stating that the differences between the levels found were rather small. However, while the fully and highly trusting individuals hardly differentiated between the levels, the means for the rather untrusting varied the most across the levels. We discuss the potential meaning behind these results. Moreover, we have expanded the discussion to highlight how our results contribute to existing theories of trust in science introduced in the theoretical chapters of the manuscript. We believe these enhancements improve the clarity and theoretical relevance of our discussion.

11. Broader Implications: While the paper addresses the immediate implications for science communication, consider expanding on broader societal and policy implications. For example, how might these findings inform efforts to build trust in science in contexts with historically low trust, such as post-colonial or non-WEIRD societies? Suggestions for future research, such as longitudinal studies to track changes in trust over time or interventions targeting specific trust dimensions (e.g., transparency, benevolence), would also provide valuable direction.

Thank you for the important suggestions. In response, we have incorporated ideas for future research, including the importance of longitudinal studies to monitor trust dynamics over time. We have also elaborated on the practical implications of our findings in the last paragraph of the discussion section by discussing possible ideas for interventions targeted at the rather untrusting individuals.

I wish the best to the authors in revising this important work.

Thank you very much for your kind and encouraging words. We genuinely appreciate your support and hope that our revisions will meet your expectations. Your positive feedback motivated us to further improve our work. Thank you once again!

**Detailed response to Reviewer #3**

Reviewer #3: This research examines trust in science during COVID-19 in South Africa, and provides typologies of individuals based on their trust levels. The use of a more comprehensive measure of trust in science is commendable.

We sincerely appreciate your encouraging comments regarding our research focus and the use of a more comprehensive measure of trust in science. Your recognition of these aspects has motivated us to further refine and develop our work.

- Paper needs to be reread thoroughly to improve the syntax in places, and typos (e.g., line 23, should read “focused on this interest”

Thank you very much for your constructive comment. We have carefully reviewed the paper to improve the syntax and correct any typographical errors, including the suggested revision on line 23. We appreciate your careful reading and helpful suggestions to enhance our quality of writing.

Methods
- Given this is a scale the authors created, it is unclear why CFA was chosen to test the dimensions of the scale. Given it is a new scale, why wasn’t EFA used?

We appreciate the reviewer’s insightful question, which has also been raised by reviewer #1. Although the previous version of the manuscript emphasised the development and validation of a new scale, we have since clarified the manuscript’s focus and removed references to scale development. Our measurement approach was primarily based on the “Public Trust in Science Scale,” an instrument based on theory that has recently been validated and published, and which closely aligns with the dimensions we examined. We have added references to this scale and its foundational instruments. This context explains our decision to employ CFA rather than EFA.

Results
- Unsure where this conclusion stems from “In sum, the fully trusting can be described as people who think that scientists have it all and who embrace science with heart and mind.”

We agree with reviewer #3 that the previous concluding statement regarding the fully trusting group was somewhat vague. To improve clarity and alignment with the measured dimensions, we have revised this description. Our intention was to create concise, memorable summaries that effectively encapsulate each group’s characteristics. In response to the reviewer’s comment, we have revisited and refined all group summaries to ensure they more accurately reflect the core traits of each group based on the five trust dimensions.

Discussion
- It would be worth discussing the generalisability of the findings in the Discussion. For instance, this sample seems very highly educated.

Thank you for highlighting this important point. As mentioned earlier, we have revised the entire discussion section and added a dedicated chapter on the study’s limitations. We fully agree that the sample’s high level of education is a key factor affecting the generalisability of our findings. To address this, we have elaborated on how the sample differs from the broader South African population and clarified that it was not fully representative. One of the main reasons for this sampling limitation was the use of an online survey conducted in English, which could not accommodate all of South Africa’s official languages.
