# Supplementary material for: Trust in science during the COVID-19 pandemic: A typology of internet users in South Africa
Source: PLoS One. 2026 Jan 28;21(1):e0340881. doi: 10.1371/journal.pone.0340881 (PMC12851501; doi:10.1371/journal.pone.0340881)
Supplement: Attachment — Submitted filename: Response_to_Reviewers_auresp_2.docx [file pone.0340881.s004.docx]

**Response to Reviewers**

Dear Reviewers, dear Editors,

We sincerely thank you for the opportunity to submit a revised version of our manuscript, entitled: “Trust in science during the COVID-19 pandemic: A typology of internet users in South Africa.” We appreciate the time and effort you have dedicated to evaluating our work, as well as the insightful comments provided.

We have carefully addressed all the comments and have thoroughly revised the relevant sections of the manuscript. We believe that these minor revisions have significantly enhanced the quality and clarity of our study.

Once again, we are grateful for your valuable and constructive feedback. We hope that, with these improvements, our manuscript now meets the journal’s standards and is suitable for publication.

Below, we provide detailed responses to each reviewer comment.

**Detailed response to Reviewer #1**

Reviewer #1: Dear Authors,
thank you for resubmitting the revised manuscript. The manuscript “Trust in science during the COVID-19 pandemic: A typology of internet users in South Africa” focuses on identifying subgroups of South African internet users with varying degrees of trust in science. Using LPA and reasons to trust science (e.g. expertise, transparency) as the basis for classification, four subgroups were identified. These subgroups were subsequently described in detail and examined regarding different forms of contact, personal experiences with science during COVID-19 pandemic, and perceived changes in trust in science.

The manuscript has benefited considerably from the revisions and now demonstrates a clear and coherent structure. All previously raised issues have been addressed satisfactorily and, where necessary, discussed in appropriate detail. I recommend the manuscript for publication, subject to the following minor adjustments:

We are pleased to read the overall positive feedback from Reviewer #1. Thank you very much for your detailed and thoughtful comments. We are happy to outline the specific revisions made in response to your suggestions below:

Content
- Optional: It may be worth considering whether the limitations could be integrated into the discussion/conclusion section, thereby allowing the manuscript to end with a concise summary or concluding remark.

We fully understand and share the reviewer's impression. As a solution, we have moved the final paragraph of the discussion chapter to the end, which now serves as the conclusion of the manuscript.

Statistics
- ANOVAs: Please provide more statistical details (i.e., F statistics, degrees of freedom, p values)

Thank you for this excellent suggestion! We have included the *F*-values, degrees of freedom (*df*), and *p*-values for all ANOVAs in the supplementary table (Table E). Additionally, we have provided the exact statistics for all *Chi²*-tests.

- Please use a capital “N” when referring to the total sample size (see, for example: Abstract l.9; full text ll. 239, 245, 279, 318, 360, 414, 420, 434, 470).

We appreciate this suggestion. However, if the editors permit, we would prefer to retain the use of “*n*” rather than “*N*”. We are aware that many authors use “*N*” in this manner, but, to the best of our knowledge, the convention is that “*N*” refers to the population, while “*n*” denotes the sample.

Miscellaneous
- L. 228 – clarification required: 18 “days”?

Thank you for pointing out where our wording was unclear. The number 18 refers to the fact that we surveyed respondents aged 18 years and older. We have slightly revised the sentence to improve clarity.

- l. 233 – “Ethics”

Thank you for identifying this error. We have corrected it.

- l. 235/236 – it might be advisable to place this statement and the corresponding reference in a footnote.

We appreciate this suggestion. However, to the best of our knowledge, the journal does not permit the use of footnotes. In response to this comment, we have made a slight adjustment to this section. We believe it will be sufficient to include information on data availability in the Additional Information section. If necessary, we are happy to revert to the original placement of this information.

- l. 645 – the first sentence should be completed.

Thank you for thoroughly reviewing the manuscript. We have now revised and completed the sentence.

**Detailed response to Reviewer #2**

Reviewer #2: The authors have responded thoroughly to my previous comments. The introduction is now clearer, with improved integration of relevant literature and explicit research objectives. The methods and results sections were strengthened by clarifying the latent profile analysis procedures, adding effect sizes, and providing richer descriptions of group differences and contact types. The discussion was reorganized to improve coherence, better connect findings to theory, and include cultural specifics and policy implications. Open-ended responses were analyzed more systematically, and limitations were more transparently discussed.

Overall, the revisions have addressed my concerns and substantially improved the manuscript. I have no further major comments.

We thank the reviewer for their positive evaluation and for acknowledging our previous revisions.

**Detailed response to Reviewer #3**

Reviewer #3: Thank you for the opportunity to review this revised manuscript. The additions to the Introduction and Discussion, and the reorganization of parts of these sections has much improved the manuscript and the intent of the research. I commend the authors on the extensive revisions to these sections.

The changes to the description of each typology is much improved. Likewise, the more nuanced discussion of the limitations of the research is a welcome addition.

We are pleased to hear that we have also convinced Reviewer #3 that our paper is ready for publication.

Thank you for clarifying the scale is based on another theoretical scale, hence the use of CFA. However, it is worth noting that this scale has been validated in a WEIRD sample, and may not necessarily translate to a non-WEIRD sample. It would be beneficial to use EFA to identity data-driven factors, and then consider the use of CFA to compare this data-driven model against the five-factor model tested. While validation of the scale is not the focus of the paper it would be useful to see this in the Supplementary Materials – or a direction for future research (i.e., cross-cultural validation of this scale). Having said that not doing so would not preclude me from recommending accept on the current version. This is just a suggestion.

Although this suggestion was not a requirement for the reviewer’s recommendation to accept our paper, we appreciated the valuable idea and incorporated it. As requested, we conducted an exploratory factor analysis, which indicated a two-factor solution. We included this two-factor model in the CFA model comparison to assess how the fit differed from our five-factor solution. Thank you once again for this helpful comment.

Typo line 135, tent should probably be “tend”.

We appreciate the reviewer’s careful reading of our manuscript. Thank you for pointing out this spelling mistake. We have revised it accordingly.

**Detailed response to Editor comments**

It is so great and speaks to the quality of the journal that the editors provided detailed feedback and suggestions what should be improved in order for the paper to be accepted. We have also taken all comments very seriously and would like to respond to each comment in detail below:

Abstract

- Please start with a sentence about what motivated the study

Thank you for this important comment. The abstract is indeed a crucial part of the manuscript, and we appreciate your suggestion. We have gladly added two introductory sentences to clarify the rationale and motivation behind the study.

- Method: where do "the five dimensions of trust in science" come from?

We hope we have understood this comment correctly. Our understanding is that it was unclear what the five dimensions were and that they were derived from theoretical work. We have now explicitly named the dimensions and clarified that they are based on theory. To our knowledge, references are usually not included in an abstract. However, if this is what you were requesting, we would be happy to incorporate it. Please let us know if we should make any additional adjustments.

- "provide clues" --> inform?

We appreciate this suggestion and have rephrased accordingly.

Introduction

- l.33 "became relevant issues" - please clarify

Thank you for pointing out the lack of clarity here. We have provided a more specific explanation, highlighting the increased public attention and visibility of scientific uncertainty and expert advice in policymaking.

- l.36-37 reference for the fragmentation

In response to this comment, we have slightly rephrased this sentence.

- What is meant by reservations - this needs more explanation, because it is presented in contrast to beliefs - which is a perception about somethings' current state (e.g, vaccine x is effective), beliefs are not attitudes (e.g., disliking vaccines)

We greatly appreciate this feedback. We have thoroughly reviewed the entire manuscript and revised all sections that may have caused misunderstandings. Both are related to beliefs - namely, believing in the promises of science and holding reservations about it.

- l.108 this study focuses on...

We have revised the section according to the suggestion.

- the paragraphs under "3 diverse types of contact with science" - please clarify the argument linking frequency of use and trust cues of certain media. At the moment pp.7-8 reads a bit back and forth

We clearly understand the critique and agree that this chapter required additional revisions. We have rearranged a few sentences to improve the flow and ensure a clearer progression of ideas, reducing the back-and-forth in the content. Thank you very much for your valuable feedback. We believe the chapter is now much improved.

- l. 219 what is the nature of trust

By nature of trust we meant theoretical types of trust in science with different patterns regarding the dimensions. Reading the manuscript again we agree that the wording was difficult to understand. This is why we have eliminated the phrase “nature of trust” in the whole manuscript – not just the suggested line – in order to explain more clearly what is meant. By “nature of trust,” we meant theoretical types of trust in science, characterised by different patterns across the dimensions. We have incorporated references to the relevant theoretical literature and directly refer to these sources when discussing our results. Upon re-reading the manuscript, we agree that this wording was somewhat unclear. Therefore, we have eliminated the phrase “nature of trust” throughout the entire manuscript (including the passage the Editor was referring to) to clarify our meaning and improve overall clarity.

- RQ3 is not very concrete, what is meant by "HOW" do they differ?

We are glad that the editor also revisited and questioned the underlying research question. Reflecting on it again, we agree that the original question was not very specific. In particular, the reasons behind the different perceived changes were missing. The research question now reads:

*RQ3:	How do groups of trust differ in the perceptions of and reasons for changes in trust in science due to the COVID-19 pandemic?*

Methods

- The power analysis is missing

Thank you for this feedback. We have now included a statement explaining how we determined the optimal sample size to ensure high power for detecting small to medium-sized differences. We assumed that the group sizes would likely vary significantly, with the smallest group potentially comprising only 5-10% of the total sample, and we anticipated up to seven groups. If this was not what the editor was asking for, we kindly ask for further clarification.

- the sample is not representative for what?

- where is the "quota plan"?

We thank the editor for their meticulous review and for upholding such high scientific standards. In the latest revisions, we have included additional information regarding the application of our quota plan and clarified how the sample deviates from the general South African public, for which the sample is not representative. The quota plan has been incorporated into a new column in Table 1.

Furthermore, upon revisiting the quota plan, we realised that we should have provided information to a different variable that measured geographical setting, as the previously reported variable was not the one to which the quota applied. Additionally, upon reviewing official South African statistics, we noticed that our sample included fewer respondents from the oldest age group than initially stated in the previous version of the manuscript. We are pleased to have identified these errors thanks to the editor’s comments.

- please provide the full questionnaire in the survey

We have now uploaded the full questionnaire to the OSF repository, where the data set and code can also be accessed.

- The first paragraph under 5.2 actually is "Procedure" and should be distinct from "Measures"

Thank you for this suggestion. We agree and have moved the paragraph to the first Methods subsection, “data collection and sampling.” We hope this meets the editor’s approval.

- What is the reference outlining the theoretical basis for the five dimensions?

In the section detailing the trust measures, we now specifically cite the papers outlining our theoretical model and the foundation for the scale. Additionally, we have slightly rearranged and rephrased this part to improve clarity and precision. We hope that it is now clearer where the five dimensions originate from.

- what is the argument for doing the CFA in the light of prior empirical tests?

The reason for conducting the CFA is that the scale is theoretically based and, similar to a newly developed scale, has so far been used only within a sample of German respondents.

- Was there a specific process for item development?

As stated, the items used to measure the five dimensions are grounded in theory and incorporate ideas from established instruments, which we have cited in the Measures section. Wherever possible, validated scales were used throughout the questionnaire. The development of the questionnaire involved an extensive process, including all authors of this paper, and it was tested, refined, and finalised through multiple feedback rounds with colleagues from South Africa. We have added this information to the Methods section.

- The item for trust assessment at the micro level needs to be discussed, scientists "in general" do not reflect individuals

This is correct. We have added a discussion in the limitations section, stating that the item measuring trust at the micro-level can be interpreted as assessing trust in scientists as representatives, and thus, trust in science at the macro level, rather than interpersonal trust in individual scientists.

- What is the internal consistency of willingsness to be vulnerable (table2)

We have added Cronbach’s alpha of the willingness to be vulnerable items to the Table as requested.

- What did coders do if they did not overlap?

Thank you for raising this important question. In cases where the two coders’ coding did not initially overlap, the cases were discussed until consensus was reached. All remaining cases were coded by a single coder. We have added this information to the Analyses section.

- Where are the summaries of the qualitative data (open responses)?

In the previous version, the open responses were included only in the dataset with the variable we used for coding. However, we believe it would be beneficial to provide an additional document with a summary of the open responses to the question analysed to address RQ3. We have now uploaded the document to the repository. Thank you very much for this excellent suggestion. It significantly enhances the transparency of the research process. The only question we have that remains is where we should refer to the materials necessary to reproduce the analyses conducted in the manuscript? Is it enough to have the link included in the Data Availability Statement?

- Could the results by affected by bad compliance (straightclickers) of the sample. What was done for the quality of the data collection?

Thank you for your valuable suggestion. We have now included this aspect in the Limitations section. Additionally, we have provided detailed information on the measures we took to ensure high data quality. Specifically, we incorporated open-ended questions throughout the survey and provided clear instructions on how to respond to each question. Many respondents used the open-ended responses to offer detailed answers, indicating a high level of engagement and attentiveness. During data cleaning, we screened the dataset for implausible response patterns. These details have been added to the Methods section. We also acknowledge in the limitations that future research should consider incorporating attention checks to further enhance data quality.

Results

- please do not write about "significant differences", either there are differences (because significant) or there could not be confirmed differences

Thank you for pointing this out. We have removed all references to "significant" differences.

- Table 3 . 4.62 (.06) -SD could be a typo here; bold indicates the "highest values"

Once again, thank you for thoroughly reviewing the manuscript. Yes, this was a typo, and we have now corrected it in the manuscript and the Appendix.

- l.457: How many used the option?

At the beginning of the results for RQ3, we have now included information on the number of respondents who provided detailed answers to the open-ended question. While all respondents answered, as the question was mandatory, more than 1,000 respondents provided responses ranging from ten words up to a maximum of 54 words. Only a few respondents (190) gave very brief answers of one to three words.

Discussion

- The argument from 534-36 to 537-39 is not clear

We have rearranged the paragraph to make the argument clearer and more comprehensible, emphasising that the results reflect the high perceived importance of aspects of scientists’ communicative abilities in relation to trust in science. Thank you for pointing out where our text lacked clarity.

- l.585-587 is too speculative

Thank you for sharing your impression. We understand your concern and have removed the overly speculative interpretation suggesting that the *rather untrusting* group primarily perceived information containing distrust cues. We hope the editors will find this revision acceptable.

- l.677/678 how many had high level of attentiveness

As stated above, the vast majority of respondents provided detailed answers to the open-ended question regarding perceived changes in trust in science due to the COVID-19 pandemic. Additionally, we have included further evidence of respondents' high level of attention in the Limitations section. Approximately 500 respondents voluntarily added comments related to the survey or the survey topic on the final page of the questionnaire.

- Please discuss: How might the groups' differential propensity for/against science may have affected responding and, so, your results.

Thank you for this valuable comment. We have added two sentences to the Limitations section addressing potential self-selection biases that may arise from surveying individuals who are voluntary members of an online access panel. These individuals might, by nature, hold more positive attitudes towards science. If you have other influences in mind regarding this issue, we would be happy to include a more detailed discussion if you could provide us with further explanation of your ideas.

- Please discuss: Was the latent profile analysis successful, if you ended up with a distinction based on the actual categories of one item?

In the Limitations section, we have also included a direct statement expressing our belief that the LPA was successful despite the addressed limitations, as meaningful patterns were identified. Most notably, while the two groups with the lowest trust in science especially emphasised scientists’ expertise, the two groups with high trust most frequently highlighted scientists’ benevolence. We interpret this as forms of *competence trust* vs. *goodwill trust*. However, we also find it an interesting observation that, empirically, we are unable to verify the existence of theoretical types of trust in science that are, for example, low in expertise and high in benevolence.

- Why do you consider high perception of negative shifts to be an artifact, why not the same about stable trust?

This is a very good question. Thank you for highlighting it! We have rearranged the paragraph to acknowledge that, in general, limitations arise from the use of self-reports and open-ended questions.

Figure 1

- Uncertainty needs to be indicated with error bars

- Capitalisation needs to be consistent

- the y-axis does not appear to be depicted

Figure 2

- Uncertainty needs to be indicated with error bars

- Capitalisation needs to be consistent

Figure 3

- Capitalisation needs to be consistent

Thank you for your valuable feedback on our figures. We have double-checked to ensure consistent capitalisation. We hope that the y-axis is now visible in Figure 1. If it is still not clear, please clarify your comment so we can address it accordingly. Additionally, we have added error bars to Figure 1 and Figure 2 to indicate the confidence intervals. Thank you again for this important suggestion.
